# Designing Studies Acceptable for Abstraction and Inclusion in Evidence-Based Laboratory Practice Guidelines

**DOI:** 10.1128/JCM.00842-18

**Published:** 2019-02-27

**Authors:** Michael A. Saubolle, Alice S. Weissfeld, Colleen S. Kraft

**Affiliations:** a Division of Infectious Diseases, Laboratory Sciences of Arizona, Banner-University Medical Center Phoenix, Phoenix, Arizona, USA; b Department of Academic Affairs, University of Arizona College of Medicine-Phoenix, Phoenix, Arizona, USA; c Microbiology Specialists Inc., Houston, Texas, USA; d Department of Pathology and Laboratory Medicine, Division of Infectious Diseases, Emory University, Atlanta, Georgia, USA; Boston Children's Hospital

**Keywords:** abstraction, clinical outcomes, evidence-based, guidelines, laboratory medicine best practices, study design

## Abstract

Expansion of technologies, changing infrastructure, and dwindling resources have produced the need for health care reform and changes in clinical laboratories. The health care model will have to shift increasingly from a fee-for-service model to a value-based model. Laboratories will have to focus more on evidence-based outcome studies evaluating the effects of their preanalytical and postanalytical practices on real patient outcomes. Although well-designed clinical trials and multicenter studies are needed to determine the effects of laboratory processes on outcomes, there has been concern that too few well-designed studies have been published. To help improve the quality of study design and to facilitate reporting transparency, several method statements have been developed. The Standards for Reporting of Diagnostic Accuracy Studies (STARD) initiative was recently updated, listing 30 items deemed crucial for transparent reporting of studies, thereby allowing the creation of a robust database for clinical practice guidelines. Three methods describing the assessment of the quality of data on which to base recommendations for such guidelines are also available. Close attention must be given to study design so that parameters ensuring study quality are met, thereby allowing inclusion of the study data in the formulation of evidence-based laboratory best practices guidelines.

## Abstract (precis)

Expansion of technologies, changing infrastructure, and dwindling resources have produced the need for health care reform and changes in clinical laboratories. The health care model will have to shift increasingly from a fee-for-service model to a value-based model.

## COMMENTARY

The practices of medicine and of clinical microbiology in the United States have been evolving because of a rapidly changing environment. Expansion of new and relatively expensive technologies, together with increasingly strained infrastructure and dwindling resources (including continued diminishing cost reimbursement), is pressing the need for health care reform. The model for health care is migrating from a fee-for-service model to a value-based model. In the new clinical environment and health care model, financial strategies of organizations will need to move away from a silo mentality of resistance to sharing information and resources and to focus on a collaborative structure in which medical care stakeholders work together to provide inclusive evidence-based best practices to ensure value in their work, as reflected in good patient outcomes (1–4). Systematic reviews of pooled data, or meta-analyses, provide quantitative measures of the overall effects of processes or testing on outcomes. Such reviews can provide strong evidence for guidelines, but only if the available studies are well designed and the sample size being studied is sufficiently large (5). Therefore, published, well-designed, clinical trials and multicenter studies are needed to determine whether specific tests or processes improve patient outcomes. The U.S. Preventive Services Task Force, which grades recommendations for clinical preventive services based on the strength of the evidence and the balance of benefits and harms, might provide a model for structuring and analyzing outcome studies.

There has been concern that too few well-designed outcome studies have been published (1–4); it is difficult to provide robust, evidence-based guidelines without systematic studies focusing on practices and their effects on clinical outcomes. To demonstrate, several recent systematic reviews and meta-analyses of available study data found that the strength of evidence ratings was frequently insufficient to allow conclusions or recommendations for or against the practices being evaluated (6, 7). In one systematic review of the literature, Buehler et al. found that 63 published studies met inclusion criteria for their review of practices to increase the timeliness of providing targeted therapy for inpatients with bloodstream infections (6). However, 47 (75%) of the 63 studies had to be excluded because study quality criteria (for example, reliability and validity of the sample population or a lack of bias) were not met. In a second review, Rubinstein et al. found that, of 812 studies considered for full-text review (of 22,207 initially screened), only 95 were eligible for inclusion in their review of the effectiveness of practices supporting appropriate laboratory test utilization (7). Of those 95 studies, 12 (13%) were further eliminated because of poor quality assessment; of the remaining 83 studies, 32 (39%) did not provide adequate information for meta-analysis in their reports.

Due to the high prevalence of poorly designed studies, methods have been proposed to help design studies to decrease their methodological flaws. These methods provide structure to the design of studies, thereby averting information being missing for several key study parameters that are crucial to the robustness of the evidence being presented and contribute to a study’s validity (8). One standard for transparency in study reporting and three methods for guidance in systematic review of evidence-based practices for inclusion in guidelines to improve patient health care outcomes have been published and are commonly used (4, 8). This commentary discusses these standards and methods to facilitate the designing and systematic evaluation of studies.

First published in 2003 and recently updated, the Standards for Reporting of Diagnostic Accuracy Studies (STARD) initiative provides a checklist describing 30 items deemed necessary for inclusion in studies and reporting (Table 1) (9, 10). Adherence to the essential items listed allows for transparency in reporting and evaluation of potential biases and sources of variability and provides the ability for readers to duplicate studies to corroborate the data being published. Estimates of accuracy in studies and processes play an important role by providing confidence in the guidance for clinical decisions and diagnostic algorithms. Compliance with the STARD initiative will allow for more robust publications and better outcomes for systematic reviews and meta-analyses in attempts to provide solid guidelines or algorithms for patient care.

**TABLE 1:** STARD 2015 list of essential items to be addressed in reporting diagnostic accuracy studies (10)^*a*^

Section or topic	Description
Title or abstract	Identification of study
Abstract	Structured summary of design, results, and conclusions
Introduction	Scientific and clinical background, objectives, and hypothesis
Methods	
Study design	Prospective or retrospective
Participants	Specific eligibility criteria, setting location, dates of study, and series type (consecutive, random, or convenience)
Test methods	Detailed description of index and reference standard tests, with rationale for choice of standard test (and any other options), definition and rationale for positivity cutoff values for index and standard tests, and blinding of test results during study
Analysis	Methods for measurement of diagnostic accuracy, handling of indeterminate results, handling of missing data, analysis of variability in diagnostic accuracy, and determination of intended sample size
Results	
Participants	Diagrammatic flow of participants, baseline demographic and clinical characteristics, distribution of severity of diseases in target condition, alternative diagnoses for subjects without target condition, and time periods and clinical interventions for both test systems
Test results	Distribution of index test results according to reference standard results, diagnostic accuracy and precision (e.g., 95% confidence intervals), and adverse effects for either test
Discussion	Limitations, sources of potential bias, statistical uncertainty, generalizability, implications for practice, intended use, and clinical role of index test
Additional information	Registration number and registry, access location for full study, and funding or support (and role of supporters)

a Adapted from reference 10.

Understanding the techniques used to assess the quality and appropriateness of study data for inclusion in clinical guidelines may be of further help to researchers for study design. Several methods have been proposed for systematic reviews of the literature and assessments of the quality of data being published. The Centers for Diseases Control and Prevention (CDC) introduced and encouraged the development of the Laboratory Medicine Best Practices (LMBP) initiative, a systematic review process to evaluate effective quality improvement practices (11). Another tool for systematic review of diagnostic practices is the Quality Assessment of Diagnostic Accuracy Studies (QUADAS), which was first published in 2003; that version has matured into an improved version called QUADAS-2 (12). A third approach to recommendations is the Grading of Recommendations Assessment, Development, and Evaluation (GRADE) (4).

The CDC LMBP initiative was developed in part to apply transparent evidence-based methods for quality improvement and to assess the effectiveness of quality improvement practices and their impacts on patient care and outcomes. The initiative developed and championed a robust A-6 methodology, which is very useful for evaluating scientific evidence in guidance development. The American Society for Microbiology (ASM) has recognized the need to provide evidence-based guidelines to allow clinical microbiology laboratories to mature in the new health care environment. In recent years, the ASM Committee on Evidence-based Medicine has partnered with the CDC and its A-6 methodology in systematically reviewing, analyzing, and appraising up-to-date published literature on the application of laboratory methods for inclusion in evidence-based laboratory practice guidelines (13). Optimization of such guidelines rests on utilization of evidence from well-designed and well-performed studies. The evidence in published studies must be systematically appraised and critically graded for the validity of results and clinical usefulness.

The value of a study is dependent on its ability to answer a clinical question being posed or evaluated. In formulating the initial question being considered in a study, a PICO strategy, consisting of (i) population, (ii) intervention or index test, (iii) comparator or reference test, and (iv) outcome, should be followed carefully. There are a number of ways to assess the quality of studies. The CDC A-6 systematic review cycle consists of six steps, i.e., (i) ask the question, (ii) acquire the evidence, (iii) appraise individual studies for inclusion, (iv) analyze the evidence, (v) apply the recommendations, and (vi) assess the impact. The third and fourth steps (appraise and analyze) focus on the evaluation of studies and the robustness of the data in them, involving screening by at least two expert reviewers. The method is based on validated evidence-based systematic review methods used in clinical medicine (11); it meets the inclusion criteria published by the National Guideline Clearinghouse (NGC) (https://www.ahrq.gov/gam/summaries/inclusion-criteria/index.html). The A-6 appraisal method provides screening criteria related to the topic, the question(s) being asked, and the practices or outcomes. The analysis rates the strength of the evidence, including study characteristics, effect size (strength of the relationships between variables), and consistency of overall quality. A quality score is given and the effect size is determined with the aid of a statistician with expertise in meta-analytical principles. The evidence data are then summarized and placed in an evidence summary table showing what information was abstracted and providing reasons why quality points were deducted.

In appraising and analyzing a study, reviewers must be able to assess what was planned, what was actually done, and what conclusions were drawn. Studies are rated on three primary sequential components of the study, i.e., its quality, its effect size(s), and its outcome measurement relevant to the review question (Table 2). Important components in a well-designed study include the sample size, assessment of the risk of bias in the identification of methods for outcome measures, and the use of appropriate statistical and analytical methods for reviewing results. Use of an adequate, well-documented sample size (number of samples studied) within a well-defined sample population (e.g., patients, sample types, or tests) is paramount. There should be a well-described method for the selection of participants or specimens (inclusion and exclusion criteria). In minimizing bias, the sample population being studied should be well defined, with documentation of which patients are included in the study (e.g., age groups), the setting described (e.g., intensive care unit [ICU] or emergency department), and the duration and time of year of the study (start and end dates). A practice being replaced with a new practice must also be carefully defined or described and a baseline delineated. Differences between groups or samples being studied should be limited to the new practice being observed. In before-after study designs, start and end dates should be clearly set for the comparator practice and the new practice being studied. In studying outcome measures, how the impact of the new practice will be measured and recorded must be defined. Good measures should be reliable, with the same outcome measures being recorded in the same way throughout the project for all practices. A good measure should be valid in accurately representing the result of the practice.

**TABLE 2:** Checklist for quality appraisal of study design (LMBP) (11)

Items for appraisal
1. Study setting
a. Study setting described adequately and documented (e.g., specific location such as ICU or emergency department)?
b. Length of study (specific start and end dates) provided?
2. Practice
a. Practice being studied described adequately, including operational requirements and components?
b. Actual duration (start and end dates) for practice documented?
3. Sample size
a. Sample population appropriately identified (what specifically is being studied)?
b. Total number of observations for sample size documented (how many are being studied)?
i. Selection criteria for study patients or specimens adequately described and documented (what was included and excluded)?
4. Comparator practice (usual or standard practice)
a. New practice studied against comparator practice or standard?
b. Major characteristics of practices (new and comparator) described in detail?
5. Outcome measures
a. Measure(s) to assess impact of new practice vs comparator practice adequately identified and defined (e.g., length of stay, time in ICU, or death)?
b. Measure(s) relevant to original question being asked?
c. Data collection method well described and documented?
6. Results
a. Final findings adequately described and supporting data provided?
b. Reported findings clearly related to practice of interest?

There are four dimensions that define study quality that reviewers rate, including (i) the study, (ii) the practice, (iii) the outcome measure, and (iv) the findings or results. Each dimension is rated separately, with each having a maximum number of points and adding up to 10 points total for each study (all four dimensions). Points are deducted for each dimension by reviewers if expected quality parameters are not met, and justification for each deduction is documented. A rating of zero in any one of four categories excludes the study from further consideration.

Dimension 1 regards the study (maximum of 3 points). The primary question asked is whether the results of the study can be generalized to other laboratories, looking at the study setting and the sample characteristics. If the study is unique to the study facility and setting, then points are deducted from its rating to gauge whether there is a likelihood that the results obtained would be achievable in other settings or laboratories. A study’s uniqueness may be in its study design, the time period, or the sample with respect to representativeness, which is a judgment regarding whether the results obtained through the study design are likely representative of the results of the practice.

Bias, defined as a systematic difference in an observed measurement from the true value, may be found in many forms. Analytical bias, methodological bias, diagnostic review bias, incorporation bias, and partial verification bias are only some forms of bias that can be introduced into studies (8). Potential bias is evaluated by focusing on the study design, the time period or study duration, and the sample itself. A rating is made regarding the extent to which the study design, period of measurement, and/or sample selection introduces possible bias into the results.

Dimension 2 represents the practice (maximum of 2 points). The intervention includes the practice being assessed. The practice should be described well enough to meaningfully distinguish it from other practices and to clarify its requirements and characteristics. Adequacy of the practice is measured through its (i) content, (ii) implementation, (iii) population/setting, (iv) training, (v) requirements, (vi) cost, and (vii) staff responsibility for practice and implementation.

Dimension 3 is the outcome (maximum of 2 points). The outcome includes the impact or effect of what is being measured. The outcome measure should capture the result of implementing the practice. Evaluation criteria for an outcome measure focus on whether a measure is valid for capturing the outcome in question. The criteria also question whether methods of recording results actually provide appropriate and accurate assessment of the practice’s impact.

Dimension 4 describes the findings or results (maximum of 3 points). Criteria for assessment of results focus on (i) sample sufficiency, (ii) appropriateness of statistical analysis, and (iii) uncontrolled deviations, along with result or conclusion biases. If the sample size is too small or the measurement period is too short to adequately capture rare occurrences as well as common occurrences, then the measure may provide an inaccurate representation of the effect of the practice. Even with common events, considerable variation may occur over time. Thus, the measurement period should be long enough to provide adequate and appropriate estimates of the practice’s impact. Appropriate statistical analysis should be used for the data accumulated.

It is evident that medicine, together with laboratory support, has evolved and will continue to evolve dramatically over this decade and the next. Evidence-based medical practice has become the central focus for diagnostic and therapeutic directions; laboratory support will require a value-added model rather than a cost-per-test reimbursement perspective. To streamline appropriate diagnostic and therapeutic approaches to achieve good patient outcomes while maintaining economic stewardship, it is important to develop evidence-based laboratory and clinical guidelines and to continually measure the continuum of care. Formulated guidelines are only as good as the study data from which they are derived. It is time to carefully reflect on the design of studies to answer specific questions and to report the results of such studies with transparency and completeness. The available methods (especially the STARD initiative and the CDC A-6 assessment tool) should be used to assess the robustness and accuracy of studies being designed, so as to provide adequate study parameters to make the reported results meaningful for developing clinical practice guidelines.
